# Sleep Disturbance in People with Anxiety or Depressive Disorders over 30 Years, and the Influence of Personality Disorder

**DOI:** 10.1080/15402002.2024.2441795

**Published:** 2024-12-16

**Authors:** Jacob D King, Min Yang, Helen Tyrer, Peter Tyrer

**Affiliations:** a Division of Psychiatry, Imperial College London, London, UK; b Faculty of Health Art and Design, Swinburne University of Technology, Melbourne, Australia; c West China School of Public Health, Sichuan University, Chengdu, China

## Abstract

**Objectives:**

Sleep disturbance is commonly reported by people with anxiety, depressive, and personality disorders, but longitudinal studies exploring the interplay of the three with disturbed sleep have not previously been described.

**Methods:**

In this study, sleep disturbance was examined among 89 patients initially presenting with anxiety or depressive disorders who provided follow-up at 12- and 30-year time points in the Nottingham Study of Neurotic Disorder. Multilevel models were used to identify factors most predictive of changes in sleep quality over time.

**Results:**

There were strong associations between poor sleep and contemporaneous severity of personality disorder and the presence of other mental disorders at 12 and 30 years follow-up, but not with disorder presence at other time points. Improvements in personality disorder were associated with improvements in sleep between time points and attenuated the positive unadjusted effects of recovery from anxiety or depressive disorders to non-significance. Relapse into further episodes of mental disorder predicted poorer sleep, whereas worsening personality disorder was not predictive of significant changes when adjusting for other factors.

**Conclusions:**

This study demonstrates the complex interplay between anxiety, depressive, and personality disorders and sleep disturbance over a long follow-up period. Future research might look to examine the relationship between personality disorder and disturbed sleep with interventional studies and by integrating personality trait research.

## Introduction

Sleep disturbance is a common feature of both anxiety and depressive disorders (Chellappa & Aeschbach, 2022; Pandi-Perumal et al., 2020). There is a well-established bi-directional relationship: anxiety and depression symptoms contribute to poor quality sleep, subjective sleep disturbance predicts the onset of depressive (Riemann & Voderholzer, 2003) and anxiety disorders (Alvaro et al., 2013; Batterham et al., 2012; Pires et al., 2016), and hinders the recovery from both (Buysse et al., 2008; Palmer & Alfano, 2020). Yet previous studies have identified several factors which confound and mediate the associations between depression or anxiety disorder diagnoses and poor sleep quality, with personality traits having long been recognized (Spielman et al., 1987). For example, high trait neuroticism and low conscientiousness are shown consistently to be associated with worse subjective sleep quality (Allen et al., 2016; Duggan et al., 2014; Stephan et al., 2018; Sutin et al., 2020), and the impact of sleep disturbance on depressive illness tentatively appears tempered when adjusting for these personality traits (Batterham et al., 2012). Correspondingly in a study among older adults, correlations between these personality factors and sleep were mediated by measures of depression (Rojo-Wissar et al., 2021).

Similarities exist in aspects of sleep quality between many psychopathological processes, notably in facets of pre-sleep hyperarousal (Baglioni et al., 2010), increased sleep onset latency, and poor sleep continuity and efficiency (Riemann & Voderholzer, 2003; Winsper et al., 2017). Meta-analysis (Baglioni et al., 2016) has failed to identify discriminatory sleep characteristics between mental health disorders, concluding instead that a transdiagnostic sleep disturbance dimension is common to many disorders (Fairholme et al., 2013).

Poor sleep quality is common among people diagnosed with personality disorder (PD) (Selby, 2013), and there appears to be short- and long-term associations with suicide ideation and attempt (Bernert & Joiner, 2007; DeShong & Tucker, 2019; Kaurin et al., 2022) making the sleep quality of people with PD a clinical priority. Increasing severity of borderline personality disorder (BPD) symptoms appears to be correlated with the severity of subjective sleep disturbance (Sansone et al., 2010), and poor sleep is associated with patients not recovering from core symptoms of BPD over time (Plante et al., 2013b). Moreover, there are strong bi-directional associations between sleep disturbance and core clinical features of both personality disorder and common mental disorders, particularly emotion regulation (Jansson-Fröjmark & Hossain, 2024; Klumpp et al., 2017; Palmer & Alfano, 2017), self-harming behaviors (Fitzpatrick et al., 2021), and drug and hazardous alcohol-use (Provencher et al., 2020). However, while sleep disturbance features as a core symptom of anxious and depressive disorders in both DSM and ICD diagnostic classifications, it is not described as a feature of personality psychopathologies despite its prevalence and likely influence in view of its importance in other mental disorders (Hafizi, 2013; McGowan & Saunders, 2021; Ruiter et al., 2012; Selby, 2013; Winsper et al., 2017).

Recent radical changes in the diagnostic system for personality disorder have aligned in the DSM-V (Alternative Model for Personality Disorders) and ICD-11, colloquially “putting ‘personality’ back into Personality Disorder” (Widiger & Hines, 2022). Recognizing that categorical models, i.e. “borderline” or “schizoid” PD, etc., had limited explanatory power failing to capture the heterogeneity of experiences of people with personality disorder, diagnosis now requires assessment on two criteria, so-called A and B. The first is an indicator of severity of personality dysfunction based on the degree of distress and impaired daily functioning related to aspects of intrapersonal (identity and agency) and interpersonal (empathy and intimacy) psychopathology. The second, is a description of the flavor of personality using a system analogous to the pathological variants of the Big 5 Personality Traits (Swales, 2022). In this sense, someone previously diagnosed with schizoid personality disorder might now be diagnosed as having “moderate personality disorder with prominent trait detachment”. Since these changes to the diagnostic system, we suggest that research into PD can be better integrated into much of the existing psychology research on personality traits, and therein offers an opportunity for better exploring the role of personality psychopathology in sleep quality and mental health disorders. Case in point, early evidence has demonstrated the associations between poor sleep and specific aspects of Criterion A, i.e., self-compassion (Brown et al., 2021; Butz & Stahlberg, 2020) within identity psychopathology, and self-regulation of emotions or behaviors (Hagger, 2010) or self-control (Liu et al., 2020) within the agency domain.

Just as anxiety and depressive disorders follow a relapsing/recurring trajectory (despite treatment) (Bai et al., 2023; Sutin et al., 2013), sleep quality is known to change over time particularly with advancing age (Li et al., 2018), and more recent evidence demonstrates how personality functioning also changes overtime often in response to changes in one’s social context and environment (Tyrer et al., 2021). In this light, a longitudinal study looking at the interplay of the three appears indicated.

While the research associating personal traits (criterion B) and sleep quality is extensive, the criteria relating to severity of personality dysfunction (criterion A) have to date, as far as we are aware, not been considered in its association with sleep quality. Furthermore, we are unaware of any longitudinal assessments looking at the association of personality disorder and sleep quality in the same individuals over time. Nor, finally, given the established associations between anxiety and depressive disorders and sleep quality, and the possible mediating role of personality traits, how the contemporary conceptualizations of personality disorder, specifically personality dysfunction, in ICD-11/DSM-V-AMPD interact with the relationship between anxiety/depressive symptoms and sleep quality. In light of the clinical need to improve the sleep quality of people with mental health disorders, our enhancing understandings of personality psychopathology and its relation to mental state disorders and sleep quality, and the existence of evidence-based treatments for personality disorder, we believe it is clinically important to explore these relationships and better understand these associations, therein potentially to highlight new treatment targets for patients with poor sleep and any of these three mental health disorders. We, therefore, aim to test the hypotheses that:

1. Sleep disturbance scores vary over a 30-year follow-up period and will be negatively predicted by age and the presence of mental disorder.
2. The severity level of personality disorder will correlate with poor sleep at each time point.
3. Improvements in sleep will be associated with recovery from anxiety or depressive disorder and improvement in personality disorder over time.
4. Trial interventions with medication, cognitive behavioral therapy or self-help administered at baseline, will not result in lasting improvements in sleep disturbance over time.

## Materials and Methods

This study is a secondary analysis of data collected from the Nottingham Study of Neurotic Disorder between 1983 and 2019. Initially, 210 participants were recruited to a three parallel-arm randomized controlled trial of 6 weeks of cognitive behavioral therapy (*n* = 84), medication (either dothiepin (*n* = 28), diazepam (*n* = 28) or placebo (*n* = 28)), or self-help interventions (*n* = 42) having presented to primary care mental health services and diagnosed with either generalized anxiety disorder, panic disorder or dysthymic disorder, or a combination of these (Tyrer et al., 1988), using a standardized interview (Spitzer et al., 1992). Alongside longitudinal assessments of mental state disorders, the study aimed to explore the implications of co-occurring personality pathology on long-term trajectory and treatment outcomes, and used assessments analogous to those used in the contemporary diagnostic criteria. Full procedural details (Tyrer et al., 1988), and results of the initial trial have been reported previously (Tyrer et al., 1993, 2021), as have reports of 12- and 30-year follow-up (Tyrer et al., 2021). Ethical approval to follow-up participants was granted by Northampton Research Ethics Committee (12/EM/0331).

These present analyses are based on 89 people from the original cohort of 210, who had complete data from baseline to 30 years of follow-up (42.8%). By this time point 71 people (33.8%) had died, and a further 50 (23.8%) were not assessed in the large part because they were no longer contactable. Attrition analyses of baseline clinical and demographic factors identified only greater age as predictive of loss to follow-up at 12- and 30-year time points. Full reporting of cohort attrition is reported elsewhere (Tyrer et al., 2021). People from all the initial treatment arms were represented in the present study’s secondary analysis (medication *n* = 32; CBT *n* = 40; self-help *n* = 17).

### Measures

The primary outcome of interest was sleep disturbance, as measured by item 4 of the observer rated Montgomery–Åsberg Depression Rating Scale (MADRS) at 12 and 30 years, and change in this sleep score between time points (Montgomery & Åsberg, 1979). Before the 12-year time-point, individual item break-down of the MADRS is not available. This item requires the clinician to assess on a 7-point Likert scale “the experience of reduced duration or depth of sleep compared to the subject’s own normal pattern when well”. Scores range from “sleeps as usual” scoring 0, through “slight difficulty dropping off to sleep or reduced, light or fitful sleep” scoring 2, “sleep reduced or broken by at least two hours” scoring 4, and sleeping for “less than two or three hours” per night scoring maximum 6.

Co-occurring mental health diagnoses were made at 12 and 30 years follow-up by Structured Clinical Interview for DSM (SCID) by a trained clinician; minor diagnoses like adjustment disorders were excluded as per a previously published approach (Tyrer et al., 2021).

Specific assessments of anxiety and depressive symptoms were made at baseline, and years 2, 12 and 30 of follow-up with the Hospital Anxiety and Depression Rating Scale’s anxiety (HADS-A) and depression (HADS-D) subscales, respectively (Zigmond & Snaith, 1983) – these scales are self-complete measures of 7 items apiece and which have been used extensively in mental health research over 40 years, and which do not enquire into sleep qualities.

The presence and severity of personality disorder was assessed by trained psychiatrists at baseline, 2, 12, and 30 years with the Personality Assessment Schedule (PAS) (Tyrer & Alexander, 1979). The PAS usually takes between 45 and 60 min to complete, and assesses 24 personality attributes on an 8-point Likert scale, and is validated to give an indication of the presence or absence of PD, and a severity level (no personality disorder, the sub-clinical “personality difficulty,” and simple, or complex personality disorder), alongside type of personality disturbance, which is analogous to, and concordant with, contemporary ICD-11 criteria (Tyrer et al., 2014). The PAS maintained good interrater agreement on example case vignettes: κ = 0.65 (Tyrer et al., 1990).

Finally, during the 12- and 30-year follow-up periods, a clinician-rated “recovery score” measuring current severity of anxiety and depressive symptoms was assessed, constructed on a 4-point scale – where 4 represents full recovery from symptoms, and 1 represents a current severe level of symptoms. A score of 4, fully recovered, was only given if there were no significant symptoms of anxiety or depressive disorders at the time of follow-up, nor contact received for mental health concerns from the person’s primary care physician or psychiatrist since the previous time-point. Further details of all measures used in this study are reported in detail in other analyses of this cohort (Tyrer et al., 1993, 2021).

### Statistical analysis

Simple correlation analyses with Spearman’s *rho* were used to identify the relationships between demographic details, personality disorder severity, diagnoses of mental health problem by DSM-III criteria, recovery from baseline anxiety or depressive disorders and sleep disturbance at each time point. Weak correlation was taken as *r* < 0.4, moderate <0.7 and strong correlation ≥0.7 (Schober et al., 2018).

Sleep disturbance at time point 12, and 30 years, as well the change (sleep score at 30 years subtract score at 12 years) in sleep disturbance over the 18-year period were the three dependent variables used in random effects models for repeated measures in a two-level (multilevel) modeling framework (which takes into account the covariance among time points by random effects at the individual level). For each dependent variable four models were conducted. Factors were entered into models in a nested approach to show possible adjustment effects of one factor on another. Model 1 examined the effects of age only. Model 2 added effects of PD severity at both earlier and current time points on current sleep disturbance. Model 3 added the presence of DSM-III diagnosis at the current time, and Model 4 added a measure of recovery from baseline anxiety or depressive illness at 30 years. For sleep change over time, the changes in PD severity and DSM-III diagnosis over the same period were entered as predictors. The independent predictive effects of personality disorder severity, DSM-III status and long-term recovery from the anxiety or depressive disorders and their association with disturbed sleep were hence estimated. In the same vein we tested how changes in PD severity and the emergence or loss of DSM-III diagnosis were associated to changes in sleep disturbance during the same period. We used the generalized Ward test to test the significance of the differences at each time point. Regression analyses were also used to test effects of initial trial allocation in the treatment group on sleep disturbance at later times. IBM SPSS v19 was used to manage the data and perform all analyses.

## Results

Female and male distribution in this cohort was 70.8% and 29.2%, respectively; there was a mean age of 30.8 ± 8.6 years at baseline. Sleep disturbance was common, with only 51.7% sleeping well (scoring 0 on MADRS item 4) at the 12-year time-point, and a minority 43.2% at 30 years follow-up. Table 1 displays descriptive statistics of key variables for the study. At 30 years, 42.7% of the cohort had fully recovered, indicating that they no longer met DSM-III diagnostic criteria for a mental disorder or those for a personality disorder under assessment by the PAS.

**Table 1.** Descriptive statistics of key variables.

Variables		Baseline	2 years	12 years	30 years
Age	Mean ± SD	30.8 ± 8.6			
DSM	Presence	89 (100.0)		43 (48.3)	45 (50.6)
	Absence			46 (51.7)	44 (49.4)
DSM change	No change			55 (61.8)	
(12–30 years)^a^	Improved			16 (18.0)	
	Worsened			18 (20.2)	
PD^b^	None	35 (39.3)	33 (37.1)	38 (42.7)	30 (33.7)
	Difficulty	16 (18.0)	16 (18.0)	16 (18.0)	16 (18.0)
	Simple	27 (30.3)	13 (14.6)	19 (21.3)	19 (21.3)
	Complex	9 (10.1)	8 (9.0)	16 (18.0)	24 (27.0)
	Missing	2 (2.2)	19 (21.3)		
PD change	No change			63 (70.8)	
(12–30 years)^c^	Improved			9 (10.1)	
	Worsened			17 (19.1)	
Initial group	Dothiepin	10 (11.2)			
	CBT	40 (44.9)			
	Self-help	17 (19.1)			
	Other drug	22 (24.7)			
Fully recovered	Yes				38 (42.7)
	No				51 (57.3)
Sleep quality score	Mean ± SD			1.47 ± 1.78	1.47 ± 1.62

DSM – Diagnosis under the Diagnostic and Statistical Manual of Mental Disorders IIIrd edition. PD – Personality Disorder.

^a^No change in DSM diagnosis is defined for those with either DSM absence or DSM presence at both 12 and 30 years; Improved DSM describes those who change from DSM presence at 12 years to DSM absence at 30 years; Worsened DSM is for those with DSM absence at 12 years changing to DSM presence at 30 years.

^b^PD severity level is also grouped into two: both no PD and PD difficulty make the negative PD group (PD+) and simple and complex PD make the positive PD (PD-) group.

^c^PD change is derived from the two PD groups at 12 and 30 years, using the same approach as for the DSM changes.

Correlation analysis reported in Table 2 demonstrates a number of findings. First, that sleep disturbance score at 12 years was moderately positively correlated with sleep disturbance 18 years later. Second, that the age of patients had no correlation with sleep disturbance at any time point. Third, while PD severity measured at baseline and the 2-year time point was not associated with disturbed sleep at any later time point, contemporary PD status had a strong positive association with sleep disturbance at the time when both were assessed. Fourth, the presence of a DSM diagnosis at 12 years was only weakly associated with also meeting criteria for a DSM-III diagnosis at 30 years (*r* = 0.24), but did have a strong and positive correlation with the time-matched PD severity level, and sleep disturbance. Recovery from anxiety and depression between 12 and 30 years’ follow-up was significantly related to improved sleep and to improved PD status.

**Table 2.** Simple correlations among sleep quality score and age, PD severity level as well as recovery score of anxiety-depression (Spearman’s coefficient rho).

	Sleep score 30 yrs	Age	PD level baseline	PD level 2 yrs	PD level 12 yrs	PD level 30 yrs	Recovery score 30 yrs	DSM_+_ 12 yrs	DSM_+_ 30 yrs
Sleep score 12 yrs	.429***	.063	.197	.173	.651***	.467***	−.342**	.581***	.447***
Sleep score 30 yrs		−.138	.172	.116	.354**	.708***	−.629***	.327**	.585***
Age			.130	.176	.063	−.282**	.218*	−.060	−.215*
PD level baseline				.355**	.265*	.289**	−.318**	.239*	.180
PD level 2 yrs					.350**	.271*	−.230	.310**	.306*
PD level 12 yrs						.420***	−.349**	.743***	.359**
PD level 30 yrs							−.710***	.393***	.839***
Recovery score 30 yrs								−.312**	−.684***
DSM_+_ 12 yrs									.236*

Two-side significance: *p < .05; **p < .01; ***p < .001. DSM – Diagnosis under the Diagnostic and Statistical Manual of Mental Disorders IIIrd edition. PD – Personality Disorder.

### Anxiety, depression, and sleep disturbance

Simple correlation analysis between anxiety and depression and sleep disturbance at 12 and 30 years demonstrated increasing strengths of associations with contemporaneousness: there were increasingly strong associations between anxiety and/or depressive scores and poor sleep at the times when both were measured. The negative relationship between sleep disturbance and recovery level over time showed a mirror image of the effect of anxiety and depression score on sleep disturbance. Given that measures of anxiety and depression were strongly correlated at all time points, to maintain the independence of covariates and to simplify the analysis only the recovery variable was used in later multivariate regression analysis as predictors of sleep disturbance.

### Effects of initial treatment allocation

Table 3 outlines the 12- and 30-year mean sleep scores of patients by their initial trial allocation arm. There was no sign of benefit from any of the initial allocated interventions on differential sleep disturbance at long-term follow-up, and patients with PD or DSM disorder presence at both 12 and 30 years had significantly worse sleep regardless of early treatment group.

**Table 3.** Sleep quality scores by initial treatment allocation and by long follow-up time points.

	Drugs		CBT		Self-help		*p* value^a^	
	PD-	PD_+_	PD-	PD_+_	PD-	PD_+_	PD or DSM	Treatments
**12 years** N mean±SD	24 1.17 ± 1.61	8 3.88 ± 1.25	34 1.24 ± 1.76	6 1.83 ± 1.84	15 0.93 ± 1.49	2 2.50 ± 0.71	**0.000**	0.637
**30 years** N mean±SD	26 0.96 ± 1.40	6 2.50 ± 1.38	25 0.72 ± 0.89	14 3.36 ± 1.50	13 1.31 ± 1.70	4 1.75 ± 1.71	**0.000**	0.994
	DSM-	DSM+	DSM-	DSM+	DSM-	DSM+		
**12 years** N mean±SD	16 0.88 ± 1.50	16 2.81 ± 1.83	19 0.26 ± 0.65	21 2.29 ± 1.90	11 0.36 ± 0.81	6 2.50 ± 1.52	**0.000**	0.280
**30 years** N mean±SD	16 0.63 ± 1.03	16 1.88 ± 1.67	17 0.41 ± 0.71	22 2.64 ± 1.62	10 0.50 ± 0.85	7 2.71 ± 1.70	**0.000**	0.731

^a^Wald test of model effects by means of linear multivariate regression analysis.

DSM – Diagnosis under the Diagnostic and Statistical Manual of Mental Disorders IIIrd edition. PD – Personality Disorder. SD – Standard Deviation.

### Predicting improvement in sleep

Table 4 presents the estimated impacts of age, PD severity, PD severity change, DSM-III diagnosis presence, DSM-III diagnosis change, and recovery status on sleep disturbance at 12 years, at 30 years, and change of sleep disturbance between 12 and 30 years, respectively.

**Table 4.** Predictive factors for improvement of sleep quality using multiple regression models.

Factors	Sleep score at 12 years		Sleep score at 30 years		Change in sleep score between 12 and 30 years^a^	
	β (SE)	P	β (SE)	P	β (SE)	P
**Model 1**						
Age	0.008 (0.026)	.767	−.031 (.020)	.133	−.043(.023)	.055
**Model 2**						
Age	−.001 (.023)	.985	−.008 (.018)	.657	−.035 (.022)	.112
PD_+_ 2 yrs	−.961 (.614)	.122				
PD_+_ 12 yrs	**2.073 (.482)**	.000	.374 (.392)	.342		
PD_+_ 30 yrs			**1.843 (.349)**	.000		
PD change(none) Improved Worsened					(Ref) **-2.35 (.592)** .023(.473)	.000 .961
**Model 3**						
Age	.004 (.019)	.817	−.001 (.017)	.960	−.035 (.021)	.098
PD_+_ 2 yrs	.075 (.542)	.891				
PD_+_ 12 yrs	.**936 (.458)**	.045	−.017 (.405)	.966		
DSM_+_ 12 yrs	**2.040 (.383)**	.000	.324 (.315)	.307		
PD_+_ 30 yrs			.**988 (0.396)**	.015		
DSM_+_ 30 yrs			**1.258 (.350)**	.001		
DSM change(none) Improved Worsened					(Ref) −.341 (.492) **1.490 (.459)**	.488 .001
**Model 4**						
Age			−.005 (.016)	.741	−.033 (.020)	.101
PD_+_ 2 yrs						
PD_+_ 12 yrs			−.001 (.384)	.997		
DSM_+_ 12 yrs			.092 (.308)	.767		
PD_+_ 30 yrs			.**957 (.376)**	.013		
DSM_+_ 30 yrs			.594 (.392)	.134		
PD change(none) Improved Worsened					(Ref) **-2.370 (.586)** −.497 (.480)	.000 .301
DSM change(none) Improved Worsened					(Ref) .017 (.467) **1.125 (.439)**	.971 .010
Recovered at 30 yrs			**-1.137 (.358)**	.002	−.718 (.378)	.057

Note: Empty cells indicate estimates not applicable.

^a^Change in sleep score is defined as (the score at 30 years – the score at 12 years). Values ranged from negative to positive, establishing a scale from improvement to worsening sleep quality over time.

DSM – Diagnosis under the Diagnostic and Statistical Manual of Mental Disorders IIIrd edition. PD – Personality Disorder.

For sleep disturbance at 12 years, meeting criteria for PD and DSM-III diagnoses at 12 years were independently associated with poor sleep: the association of a DSM diagnosis with poor sleep quality was stronger than that of a positive diagnosis of PD. The age of patients had no impact, nor did previous PD and DSM diagnoses at the 2-year follow-up point predict sleep disturbance 10 years later. Likewise, age, PD, and DSM diagnosis at 12 years did not show significant impacts on sleep disturbance at 30 years, but a PD or DSM diagnosis at 30 years was significantly and independently associated with poorer sleep at 30 years. The impact of a DSM-III diagnosis was attenuated by the effect of recovery from anxiety or depressive disorders at 30 years while they themselves predicted significant improvements in sleep disturbance. The strong negative predictive effect of PD status on sleep disturbance at 30 years was resistant to controlling for the benefit of recovery from anxiety or depression.

In relation to sleep disturbance change over 18 years (from the 12-year to 30-year follow-up points), age alone had a small negative effect which was weakened by the strong effect of improving PD severity. The same finding was evidenced by the relationship between changes in sleep disturbance and the presence of DSM-III diagnosis, showing that developing a new DSM-III diagnosis was significantly associated with worsening sleep over time. The effect of recovery on change of sleep disturbance was small, which did not affect the co-varied relationship between PD or DSM changes and change in sleep disturbance.

## Discussion

This study highlights the associations of anxiety, depression and personality disorders with sleep over a long follow-up period. The presence of sleep disturbance was high in this population. For comparison, a study using the same method of assessment of sleep disturbance in a similarly aged group of individuals from a general population sample in Sweden (mean age 67.5, s.d 7.9) reported sleep disturbance in only 19.7%, compared with 56.8% in the present cohort at the 30-year follow-up period (mean age 60.8, s.d 8.6) (Sindi et al., 2020).

There were two notable and novel findings from this analysis. First, because we used an approach of personality assessment analogous to ICD-11 Personality Disorder severity assessment, these results were able to demonstrate a gradient between increasing severity of personality disorder and poorer sleep duration and depth in the same individuals at different stages of their life. To date, work on this topic had used cross-sectional methods or dichotomous PD outcome measures (Plante et al., 2013b; Sansone et al., 2010). Moreover, when controlling for contemporary personality disorder severity, personality disorder presence at other time points was not associated with sleep disturbance, suggesting that the degree of sleep disturbance moves in-step with severity of personality pathology across the life course. Together, these observations highlight the role of poor sleep as an independent indicator of current personality disorder severity, rather than there being a persistent longitudinal effect of a historical personality disorder diagnosis. In the McClean Study of Adult Development, an American longitudinal study of 290 people with (severe Borderline) personality disorder, at the 16-year follow-up mark, patients who reported disturbed sleep were significantly less likely to have achieved recovery from their BPD symptoms (Plante et al., 2013b). Authors of that study had concluded that poor sleep may, therefore, hamper recovery from BPD. Our study builds on this by demonstrating changes in sleep disturbance follow in-step with personality dysfunction severity. We might, however, suggest that while both studies show that sleep disturbance may indicate contemporary personality dysfunction, the mediation pathway remains unclear: does personality decompensation lead to poor sleep as a symptom (among others) of poor personality functioning, does poor sleep in part perpetuate personality decompensation, or do factors which both precipitate sleep disturbance also precipitate mental health “crisis”?

Second, these results also demonstrate that poor sleep is positively associated with the severity of personality disorder independent of co-occurring anxiety or depressive illness. While unadjusted models confirm pre-established findings that recovery from mental illness is associated with improved sleep outcomes, the present results suggest that these benefits are associated instead with improvements in co-occurring personality dysfunction. Correspondingly, recovery from anxiety and depressive disorders was not associated with improvement in sleep disturbance as might have been expected when improvements in personality functioning were taken in to account. The recurrence or development of a new mental state disorder, however, was shown to be strongly associated with a worsening sleep disturbance, while worsening personality functioning was not. These findings suggest that the previously conceptualized bi-directional relationship of anxiety and depressive disorders and sleep is more complex than currently understood, and challenge the notion that treating depressive or anxiety disorders without addressing personality functioning will improve sleep. In this way, findings mirror those which indicate personality factors have a complex association in the relationship between common mental disorders and sleep quality (Duggan et al., 2014; Rojo-Wissar et al., 2021).

There are several mechanisms by which sleep disturbance is linked with personality disorder, but none has achieved wide-spread acceptability in the literature. Some evidence has pointed to a shared etiology from temperament, childhood adversity, maladaptive parenting, nightmares, other primary sleep disorders, hypothalamic–pituitary axis dysfunction (Lereya et al., 2017), and circadian rhythm disturbance (McGowan & Saunders, 2021). Other authors highlight causative or mediating factors of PD which contribute to disturbed sleep including drug and alcohol use, emotional dysregulation (Simor & Horváth, 2013), or cognitive distortions (Plante et al., 2013a). Similarly, reverse causation is postulated given that disturbed sleep is associated with emotional lability, impulsivity, and self-harming behaviors (Winsper & Tang, 2014).

In light of this study’s findings that an improving PD function is associated with improved sleep, we propose that addressing personality dysfunction with established interventions could lead to improved sleep for patients resistant to other approaches. For example, by improving those facets of personality dysfunction shown tentatively already to be associated with sleep quality (i.e. self-worth/compassion, emotion and behavior regulation, and capacity for self-direction) (Brown et al., 2021; Hagger, 2010; Liu et al., 2020). Moreover, the reverse may also stand, and improving sleep by targeted intervention could herein have benefit to the core outcomes of those with personality disorder, as it is beginning to be shown in many mental disorders (Cunningham & Shapiro, 2018), as well as chronic pain (McCrae et al., 2019) and fibromyalgia (Climent-Sanz et al., 2021). Experimental studies which improve sleep quality with established interventions (i.e. psychological and pharmaceutical), and measure personality disorder severity and associated outcomes as well as that which integrates research on personality trait profiles and co-occurring mental state disorders would be of benefit to clarify this relationship.

### Limitations

The main limitation of this study was that the outcome measure for sleep disturbance was a single item, and while items in the MADRS are constructed on Likert scales and treated as continuous variables (Quilty et al., 2013) these individual Likert-type items have not been assessed for their construct validity, or suitability for assessment of a unifying concept as a continuous variable. Moreover, this item is based on observer judgment rather than objective measures of sleep quality, and relates only to aspects of duration and depth. Additionally, it would have improved the study if sleep quality was available at more time-points, particularly baseline, and as the time points available may not be the most optimal for capturing changes in sleep, mental state and personality profiles, and naturally more frequent assessments would give a sharper image of the trajectory of these variables. In this study, we grouped participants with all baseline depressive or anxiety disorder diagnoses together, thus we cannot make judgment on the differential effects of baseline diagnosis on sleep trajectory.

It would be pertinent for future studies of sleep disturbance in personality and mental state disorders to use validated assessment scales or objective outcomes which assess multiple facets of sleep quality. In addition, given the complexity of the relationship between anxiety, depressive and personality disorders, and sleep disturbance, there are several key confounders through the timeline for which we have not been able to account, particularly current medication (especially sedative and hypnotic agents), drug and alcohol use, major life events, primary sleep disorders, and physical health comorbidities.

## Conclusions

Sleep disturbance may be a “neglected symptom” (Simor & Horváth, 2013) of personality disorder, given that these results have shown a strong, temporal, and graded relationship. Recent developments in diagnostic criteria for personality disorder better facilitate work which might now look to include personality traits, and severity of personality dysfunction into the established literature base tackling the links between common mental health disorders and sleep. Future work in this field may explore whether sleep disturbance can be used as a severity indicator of personal disorder, or indeed whether improving sleep quality improves core outcomes of personality disorder and *vice versa*.

## Data Availability

Data relating to these analyses are available from the corresponding author on reasonable request.
